# Recalled Parental Emotion Socialisation and Psychological Distress: The Role of Emotional Schemas

**DOI:** 10.1177/00332941231204304

**Published:** 2023-09-28

**Authors:** Rita Sebastião, David Dias Neto, Ana Nunes da Silva

**Affiliations:** School of Psychology, ISPA—Instituto Universitário, APPsyCI—Applied Psychology Research Center Capabilities & Inclusion, Lisbon, Portugal; Faculdade de Psicologia, CICPSI - Centro de Investigação Em Ciência Psicológica, Universidade de Lisboa, Lisbon, Portugal

**Keywords:** Beliefs about emotions, emotional invalidation, emotional schemas, parental emotion socialisation, psychological distress

## Abstract

Recalled parental emotion socialisation has been associated with psychological distress in adulthood. Since emotional schemas develop from interpersonal interaction and can result in pathological reactions, they can be an important mechanism. The present study analyses the mediator role of emotional schemas in the relationship between recalled parental emotion socialisation and psychological distress. A community sample of 246 Portuguese adults, between 18 and 73 years old (*M* = 34.3, *SD* = 13.32), completed the following self-report measures: Emotional (In)Validation Experiences Scale; Leahy Emotional Schema Scale; and Brief Symptom Inventory 18. Two mediation models were estimated, one for each parent. Emotional schemas mediated the relationship between recalled parental emotion socialisation and psychological distress. Negative evaluation of emotion was the strongest mediator in the relationship between emotional invalidation and psychological distress while difficulties in reappraisal was the strongest mediator in the relationship between emotional validation and psychological distress. Differences were observed between the mother’s and father’s models. Results highlight the importance of emotional schemas in understanding the role of parental emotion socialisation in psychological distress, which may have important implications for intervention and prevention.

## Introduction

Parental emotion socialisation is the set of parental behaviours that influence children’s learning about emotion expression, regulation, and experience (Eisenberg et al., 1998). This important dimension of parenting has been associated with psychopathological symptoms in adulthood (Boucher et al., 2013; Garside & Klimes-Dougan, 2002; McKee et al., 2019). Although parental emotion socialisation was related to the development of psychosocial functioning, understanding the mechanisms that contribute to explain the role of recalled childhood and adolescent experiences in psychological distress is not fully explored, and it is critical and relevant for improving clinical intervention.

### Parental Emotion Socialisation and Psychological Distress

Parental emotion socialisation can occur through contingent behaviour, modelling, or formal instruction (Edwards & Wupperman, 2018; Eisenberg et al., 1998). Contingent behaviour concerns how parents typically respond to their children’s emotions (e.g., validating vs. invalidating responses) (Eisenberg et al., 1996; Fabes et al., 1990, 2002). Modelling can occur when parents show their emotions (Eisenberg et al., 2003; Halberstadt et al., 1995). Finally, formal instruction refers to parents' emotional coaching of their children (Gottman & DeClaire, 1997; Gottman et al., 1996). These different ways of parental emotion socialisation result in emotional validation or invalidation experiences. Emotional validation experiences occur when parents support their children’s emotional expression, know how to teach, guide their emotional experience, and adaptively express their emotions. Emotional invalidation happens when a parent punishes, minimises, or demonstrates the unacceptability of an emotional expression.

Recalled parental emotional invalidation is found to be associated with psychopathological symptoms in adulthood. It has also been linked to anxiety, depressive, borderline, and schizotypal personality symptoms, alexithymia, and psychological distress (Arseneault, 2013; Boucher et al., 2013; Dinis, 2014; Faro et al., 2019; Garside & Klimes-Dougan, 2002; McKee et al., 2019; Ramakrishnan et al., 2019; Sauer & Baer, 2009, 2010). On the other hand, the literature has associated recalled parental emotional validation with better mental health in adulthood. Some studies (McKee et al., 2019; Sauer & Baer, 2009, 2010) observed that recalled parental emotional validation was associated with less anxiety, depression, and borderline personality symptoms.

Both parents converge in socialising their children’s emotions in many aspects, but there may be some differences (Brand & Klimes-Dougan, 2010; Faro et al., 2019). Studies observing the differential role of mother and father suggest different functions. Ramirez (2018) found that recalled paternal emotional invalidation, but not maternal, was associated with anxiety symptoms. In the case of depressive symptoms, maternal and paternal emotional invalidation was linked to these symptoms. Lugo-Candelas et al. (2016) compared the role of mother and father recalled socialisation practices. They observed that fathers’ socialisation was associated with emerging adults’ functioning across ethnic groups. However, the relationship between mothers’ socialisation and emerging adults’ functioning varied across ethnic groups. Specifically, recalled maternal emotional invalidation was related to anxiety, hyperactivity, attention deficit, oppositional disorder symptoms, and alcohol use and abuse only in Euro-American and Afro-American families. These differences can be explained by different sample characteristics, such as age, gender, and culture, since these variables influence parental emotion socialisation (Eisenberg et al., 1998). However, these studies suggest a differential role of mother and father emotional socialisation on the development of psychological distress.

### Parental Emotion Socialisation and Emotional Schemas

Childhood and adolescent experiences in the interpersonal context seem to be particularly important in influencing the interpretation of emotions (Leahy, 2019). It has been proposed that emotional schemas are based on experiences and interactions with significant others. People learn about emotions by observing how their parents and significant others react and express emotions (Leahy, 2019). Thus, parental emotion socialisation influences the development of emotional schemas that may permeate interpersonal functioning and emotion regulation throughout life (Leahy, 2019). Independently on how parental emotional socialisation occurs, it influences the processing of information related to emotions (Eisenberg et al., 1998), the development of adequate emotional competencies, emotional regulation abilities (Gross & Jazaieri, 2014), coping abilities (Hersh & Hussong, 2009) and development of emotional schemas (Leahy, 2019).

The emotional schemas model is recent, so relatively little empirical attention was given to this developmental perspective (Edwards & Wupperman, 2018). Nevertheless, some studies (e.g., Krause et al., 2003; Sauer & Baer, 2009, 2010) suggest a relationship between recalled parental emotional invalidation and specific maladaptive emotional schemas, such as the fear of losing control of emotion and rumination. More specifically, Dinis (2014) observed that recalled parental emotional invalidation was associated with maladaptive emotional schemas and negatively related to adaptive emotional schemas. Parental emotional validation was associated with adaptive emotional schemas and negatively related to maladaptive emotional schemas. In this regard, experiences of being ignored, criticised, humiliated, dismissed, or minimised appear to have a lasting effect on individuals’ beliefs about their emotions and how others will respond (Edwards et al., 2016; Leahy, 2015, 2019).

### Emotional Schemas and Psychological Distress

According to the emotional schema model (Leahy, 2002), these are central to psycho-emotional functioning more than the emotions themselves. Emotional schemas are active when humans experience an emotion (Leahy, 2002, 2012). It involves individual-specific beliefs about emotional experiences (Edwards & Wupperman, 2018; Leahy, 2015). It may include beliefs, such as the typical course of emotion, causes and consequences of emotion, implications of emotional experience on self-concept, and means of regulation (Leahy, 2002).

Maladaptive emotional schemas that are self-judgmental, rigid, and avoidant of emotions are associated with poorer psycho-emotional functioning and can cause and maintain mental health problems (Edwards et al., 2016; Edwards & Wupperman, 2018; Leahy, 2015). Several studies have shown that maladaptive emotional schemas are present in depression (Batmaz & Özdel, 2015; da Silva et al., 2022; Leahy, 2002; Leahy et al., 2012; Sirota et al., 2016, 2018), anxiety (da Silva et al., 2022; Dinis, 2014; Leahy et al., 2018; Sirota et al., 2016, 2018; Suh et al., 2019; Tirch et al., 2012), obsessive-compulsive disorder (Oguz et al., 2019; Sirota et al., 2016, 2018), alcohol use and dependence (Leahy et al., 2011; Suh et al., 2019), eating disorders (Suh et al., 2019), and paranoid ideation, somatic and psychotic symptoms (da Silva et al., 2022).

### The Role of Emotional Schemas

Emotional schemas develop from interpersonal interaction during childhood and adolescence (Leahy, 2019). It guides the interpretation of emotional information that can result in pathological reactions (da Silva et al., 2022; Leahy et al., 2018; Suh et al., 2019). Thus, they can play a role in the relationship between parental emotion socialisation and psychological distress. The interrelation of these three concepts has not been studied so far. However, some studies already investigated the role of similar variables. For example, Krause et al. (2003) conducted a study in a community sample and found that emotional inhibition mediated the relationship between emotional invalidation in childhood and anxiety and depressive symptoms in adulthood. Emotional inhibition was assessed through the following dimensions: rumination, a tendency to suppress, avoid and retain unwanted thoughts, avoidance coping, and avoidance reactions to a specific stressful event. Boucher et al. (2013) observed that the attitudes towards sadness mediated the association of parental emotion socialisation with depression in adulthood in college students. These can be understood as specific emotional schemas for sadness. For example, “fear of being rejected if sad” and “fear of where sadness might lead” refer to specific emotional schemas. Westphal et al. (2016), based on a clinical sample, found that general parental indifference during childhood (i.e., not specific to emotions) predicted adult borderline personality, depression symptoms, and post-traumatic stress disorder through the mediation of invalidation (a specific emotional schema). In this regard, Sauer and Baer (2009), in a college student sample, observed that the association of invalidation environmental in childhood on borderline personality symptoms was mediated by the fear of losing control of emotions. In the case of alexithymia, Edwards et al. (2016) found that emotional schemas mediated the relationship between emotional invalidation and later alexithymia. Additionally, Dinis (2014), in a clinical sample, found that the association of emotional invalidation on anxiety and depressive symptoms occurs through maladaptive emotional schemas. In sum, the relationship between parental variables (e.g., parental indifference, parental invalidation) and psychological distress seems to be explained by emotional schemas or similar variables within different studies.

### The Current Study

**Figure 1.**
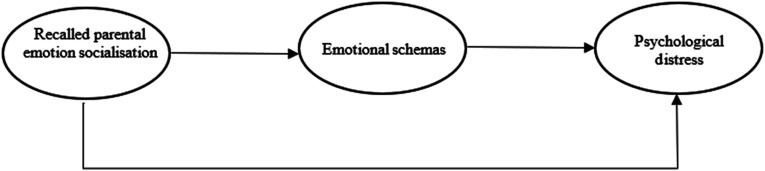
The hypothesised model – one for each parent.

Although the relationship between recalled parental emotion socialisation and psychological distress in adulthood has been studied, the processes that underlie this relationship are still under debate. Knowing what past dimensions are relevant to emotional functioning is important in understanding how these dimensions are linked to psychological distress. The main objective of the study is to contribute to understanding the role of emotional schemas in the relationship between recalled maternal and paternal emotion socialisation and psychological distress in adulthood. This objective is achieved by contrasting the role of each parental figure. Thus, the present study aims to test two mediation models to evaluate the mediator role of emotional schemas on the relationship between recalled parental emotion socialisation and psychological distress in adulthood (H1). This model proposes that recalled parental emotion socialisation predicts emotional schemas in adults (H2), which, in turn, predicts psychological distress (H3). An association between parental emotion socialisation and psychological distress is expected (H4). The hypothesised model is summarised in Figure 1.

## Method

### Participants

This study was conducted in Portugal, with a convenience sample of 257 adults. Two of these declined to participate, and nine were excluded because they did not complete their questionnaires. The final sample included 246 participants between 18 and 73 years old (*M* = 34.3, *SD* = 13.32). 208 (85%) participants were female. Most participants were single, held a BA, worked full time, and did not report having any diagnosed mental disorder. Most participants lived in their original family during the first eighteen years of life, did not suffer the death of either parent (92%), or experienced the parents’ divorce (83%). For a more detailed analysis of the sample’s characteristics, see Table 1.

**Table 1.** Sample Characterisation (*n* = 246).

Variable	Characterisation
Marital status	Single 139 (56.5%); married 80 (32.5%); divorced 23 (9.3%); widowed 4 (1.6%)
Level of education	Six years 1 (.4%); nine years 13 (5.3%); twelve years 34 (13.8%); Bachelor’s degree 155 (63%); Master’s degree or superior 43 (17.5%)
Professional status	Full-time employee 134 (54.5%); part-time worker 10 (4.1%); unemployed 13 (5.3%); student 63 (25.6%); retired 5 (2%); others 21 (8.5%)
Mental disorder diagnosed	Yes 18 (7.3%); No 224 (91.1%); prefer not to answer/I don’t know 4 (1.6%)
Family (of origin) type	Single parent 25 (10.2%); original family 208 (84.6%); reconstructed family 10 (4.1%); other types 3 (1.2%)

### Measures

Recalled parental emotion socialisation was evaluated by the Emotional (In)Validation Experiences Scale (EVES; Dinis & Gouveia, 2011). This questionnaire assesses perceptions of emotional validation or invalidation of each parent (or substitutes) during the first eighteen years of life, with a five-point Likert scale (1 = *never true* to 5 = *always true*). The questionnaire consists of two dimensions: (1) Emotional Validation Experiences that reflect a parent that validates and supports the children’s emotions, including eight items. Higher scores reflect more validation experiences; (2) Emotional Invalidation Experiences that refer to a parent that punishes, minimises, or demonstrates unacceptability to the emotional expression, including 13 items. Higher scores reflect more invalidation experiences. EVES simultaneously evaluates parental emotion socialisation through contingent behaviour, modelling, or formal instruction. The measure has shown good internal consistency in each subscale (α ≥ .89) (Dinis, 2014; Dinis & Gouveia, 2011). In the present study, it showed a very good internal consistency (α ≥ .92).

Emotional Schemas were assessed by the Portuguese version of the Leahy Emotional Schema Scale (LESS; da Silva et al., 2022; Leahy, 2002). It is a 42-item scale consisting of statements on how individuals cope with their beliefs and emotions about their own emotions. Participants rate each item on a six-point Likert scale (1 = *very untrue of me* to 6 = *very true of me*). Items can be grouped into five components: (1) Negative Evaluation of Emotion, which has several items that reflect some type of control, incomprehension, or non-acceptance. Some items seem also to reflect a secondary emotion towards the emotion felt; (2) Difficulties in Reappraisal, seem to refer to a dimension of cognitive emotion regulation, suggesting a reappraisal dimension; (3) Difficulties in Naturalising Emotion, not only capturing a non-acceptance of what the individual is feeling but also that he doesn’t feel the same as other human beings; (4) Need to Be Rational, the content of the items reflect not only the need to be rational but also a devalue of emotions; (5) Simplistic View of Emotion, related to a simplistic view of emotion and rumination dimension. Higher scores reflect more maladaptive emotional schemas. LESS has shown adequate to very good internal consistency - from ω = .66 to ω = .93 (da Silva et al., 2022). In the present study, the scale demonstrated acceptable to very good internal consistency (from α = .60 to α = .94).

The Brief Symptom Inventory 18 (BSI-18; Canavarro et al., 2017) was administered to assess psychological distress. This questionnaire corresponds to the Portuguese adaptation of the Brief Symptom Inventory 18 (Derogatis, 2001), evaluating psychological malaise in the prior week, with a five-point Likert scale (0 = *not at all* to 4 = *extremely*). It comprises three symptom scales: Somatisation, Depression, and Anxiety. Scores on the total of items are summarised on the Global Severity Index (GSI), which corresponds to a measure of overall psychological distress level, with higher scores reflecting more significant psychological distress (Canavarro et al., 2017; Nazaré et al., 2017). The BSI shows alphas between .80 and .93 in a clinical and community sample respectively (Canavarro et al., 2017; Nazaré et al., 2017). In the present study, the three subscales and GSI displayed good and very good internal consistency (from α = .84 to α = .93).

### Procedure and Data Analysis

Data collection for this cross-sectional study was conducted online through an anonymous survey distributed through social media (e.g., Facebook, Instagram) and social institutions (e.g., schools). Data collection followed this approach to include people who might not otherwise have access to the questionnaire, ensuring a diverse sample in terms of parental emotional socialisation experiences.

The Ethical Principles of Psychologists and Code of Conduct were abided by, and the data was collected. After consent was obtained, all participants completed the self-report measures, including demographic information, the EVES (Dinis & Gouveia, 2011), the LESS (da Silva et al., 2022), and the BSI-18 (Canavarro et al., 2017).

After data collection, we analysed it through the software Statistical Package for the Social Sciences 25 (SPSS; IBM Corp, 2017). The considered significance level of all analyses was .05. The structural Equations Model (SEM) was used to study whether emotional schemas mediated the relationship between recalled parental emotion socialisation and psychological distress. Two different models were considered - one for each parental figure. Sociodemographic factors (sex and age) were controlled in the structural model. Items with factor loadings <.4 were removed from the model (Marôco, 2021). They were fitted with the lavaan package (Rosseel, 2012) for the R statistical system. The estimation was carried out with Diagonally Weighted Least Squares (DWLS) estimation. The goodness of fit for the model was assessed through the Chi-square statistics, Comparative Fit Index (CFI), Tucker-Lewis Index (TLI), Root Mean Square Error of Approximation (RMSEA), and Standardized Root Mean Squared Residual (SRMR). The fit was judged adequate for CFI and TLI above .9 and RMSEA and SRMR below .08 (Marôco, 2021).

## Results

The participants showed a mean of 1.91 (*SD* = .90) regarding the father’s recalled emotional invalidation experiences and 1.99 (*SD* = .88) regarding the mother’s emotional invalidation experiences. Additionally, participants reported 2.67 (*SD* = 1.30) in the father’s emotional validation experiences and 3.17 (*SD* = 1.15) in the mother’s emotional validation experiences. This is similar to other studied community samples (Dinis, 2014). Regarding emotional schemas, negative evaluation of emotion showed mean scores of 2.75 (*SD* = 1.09), difficulties in reappraisal averaged 2.79 (*SD* = .95), difficulties in naturalising emotion showed mean scores of 2.88 (*SD* = 1.00), need to be rational averaged 3.17 (*SD* = .83) and simplistic view of emotion showed mean scores of 4.40 (*SD* = 1.18). These results are close to the means found in the adaptation of the scale for the Portuguese population (da Silva et al., 2022). Finally, concerning psychological distress, participants presented a mean score of 20.18 (*SD* = 14.43). This result is slightly higher than the mean found in the adaptation of the scale for the Portuguese population in the community sample (Nazaré et al., 2017).

Two models (one for each parent) assessing the mediation role of emotional schemas dimensions in the relationship between recalled parental emotion socialisation and psychological distress were tested, controlling sociodemographic factors.

Regarding the father model, the proposed model showed a good fit to the data (*χ*2 (2807) = 3953.93, *p* < .001, CFI = .94, TLI = .94, RMSEA = .04, *p* = 1.00, SRMR = .076). No modification indices were used to improve fit. Negative evaluation of emotion and need to be rational were significant mediators in the relationship between recalled emotion socialisation (validation and invalidation) and psychological distress (*β*_invalidation-negative evaluation-psychological distress_ = .24, *SE* = .06, *p* = .004; *β*_validation-negative evaluation-psychological distress_ = −.15, *SE* = .04, *p* = .011; *β*_invalidation-need to be rational-psychological distress_ = −.08, *SE* = .02, *p* = .023; *β*_validation-need to be rational-psychological distress_ = .06, *SE* = .02, *p* = .032). Difficulties in reappraisal was a significant mediator in the relationship between recalled emotion validation and psychological distress (*β* = −.19, *SE* = .04, *p* = .002). The total standardised effect of recalled emotional invalidation on psychological distress was .37 (*SE* = .05, *p* < .001), and of recalled emotional validation on psychological distress was −.20 (*SE* = .04, *p* = .002). Overall, the model explained 23.5% of the negative evaluation of emotion, 24.4% of the difficulties in reappraisal, 14.8% of the difficulties in naturalising emotion, 14.3% of the need to be rational, 7.1% of the simplistic view of emotion and 60.3% of the psychological distress.

Regarding the mother model, the proposed model showed a good fit to the data (χ2 (2807) = 3997.41, *p* < .001, CFI = .93 TLI = .93, RMSEA = .04, *p* = 1.00, SRMR = .076). No modification indices were used to improve fit. The negative evaluation of emotion was a significant mediator in the relationship between recalled emotion socialisation (validation and invalidation) and psychological distress (*β*_invalidation-negative evaluation-psychological distress_ = .22, *SE* = .05, *p* = .007; *β*_validation-negative evaluation-psychological distress_ = −.11, *SE* = .04, *p* = .041). Difficulties in reappraisal was a significant mediator in the relationship between recalled emotion validation and psychological distress (*β* = −.18, *SE* = .04, *p* = .003). The total standardised effect of recalled emotional invalidation on psychological distress was .30 (*SE* = .05, *p* < .001), and of recalled emotional validation on psychological distress was −.11 (*SE* = .04, *p* = .098). Given that the indirect effects differed in valence from the direct effect, they can suppress each other, resulting in total effect in smaller or non-significant effects (MacKinnon et al., 2000).

Overall, the model explained 18.1% of the negative evaluation of emotion, 23.6% of the difficulties in reappraisal, 9.1% of the difficulties in naturalising emotion, 11.4% of the need to be rational, 7.9% of the simplistic view of emotion and 59.1% of the psychological distress.

**Figure 2.**
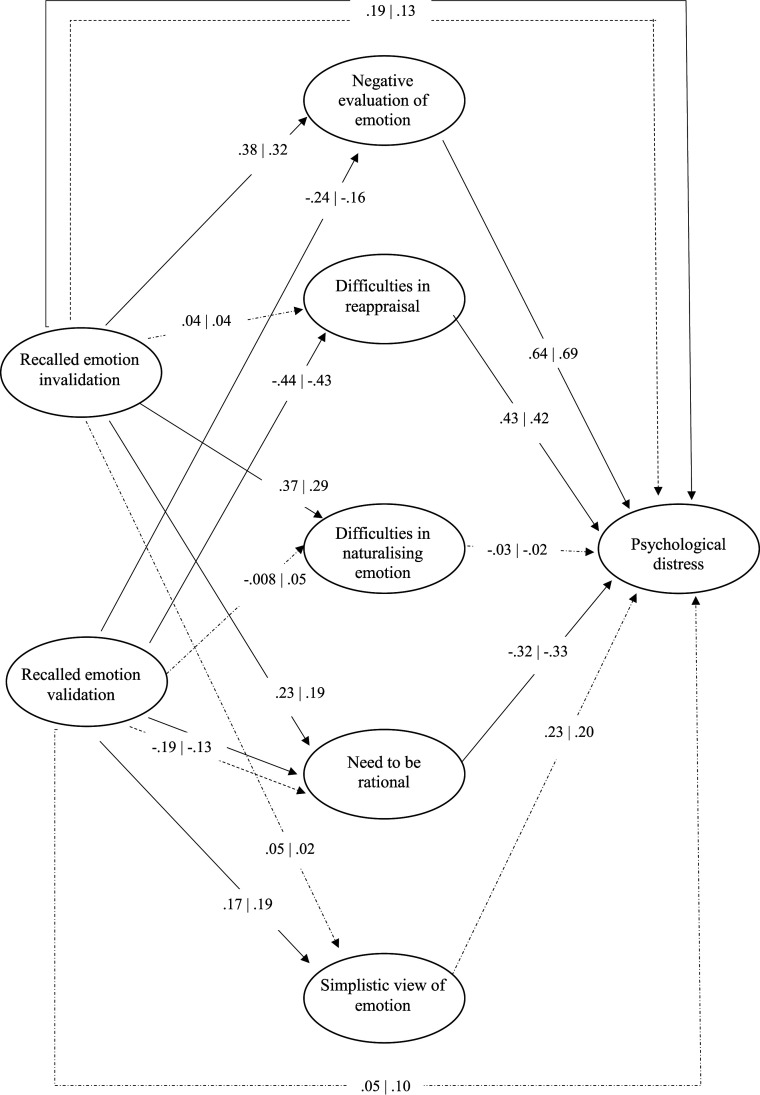
Standardised estimates of regression coefficients for the structural Father’s and Mother’s models. Note. Dashed lines represent non-significant relationships between variables. Coefficients on the left are from father’s model | Coefficients on the right are from mother’s model.

Figure 2 shows the results of the father’s and mother’s structural models, and Table S1 presents the total, direct, and indirect effects of the final models for each parental figure. Both models demonstrated a positive effect of the negative evaluation of emotion and difficulties in reappraisal and a negative effect of the need to be rational on psychological distress. There was a positive effect of recalled emotional invalidation on negative evaluation of emotion, difficulties in naturalising emotion, and the need to be rational. And there was a negative effect of recalled emotion validation on negative evaluation of emotion, difficulties in reappraisal, and a positive effect of recalled emotion validation on simplistic view of emotion. In the father model, recalled emotional validation had a negative effect on the need to be rational. Furthermore, paternal emotional invalidation continues to relate directly to psychological distress in adulthood.

## Discussion

The main aim of the present study was to understand the role of emotional schemas in the relationship between recalled parental emotion socialisation and psychological distress. Using SEM, we analysed the mediation role of emotional schemas for each parental figure. The hypotheses were supported in both models. The findings suggest that individuals who reported more parental emotional invalidation present more (maladaptive) emotional schemas and report more psychological distress. On the other hand, participants who remembered more parental emotional validation presented less (maladaptive) emotional schemas and reported less psychological distress.

These results are consistent with previous research (Boucher et al., 2013; Dinis, 2014; Edwards et al., 2016; Krause et al., 2003; Sauer & Baer, 2009; Westphal et al., 2016). They support the idea that individuals who have grown up with invalidating parents evaluate their emotions more negatively, perceiving them as incomprehensible, uncontrollable, unacceptable, and shameful. This evaluation may contribute to the development of psychological distress. In contrast, individuals who have grown up with parents who mostly validate and support their emotions tend to do less self-judgmental, rigid, and avoidant evaluations of emotions. This evaluation, in turn, is associated with less psychological distress.

However, some exceptions were observed. Parental emotional validation was positively associated with a simplistic view of emotion. This is an unexpected result since a simplistic view of emotion corresponds to a limitation in the ability to understand that one can have conflicting and complicated feelings about self and others (da Silva et al., 2022). This could be explained by the tendency of emotional validation to be centred on expressing positive emotions, which tend to be less ambivalent or have less complexity in the relationship between intervenients. Nonetheless, the simplistic view of emotion was not a significant mediator in either of the models. Additionally, in both models, the need to be rational was negatively associated with psychological distress. Paternal emotion socialisation contributed to the development of the need to be rational, and it can be seen as having a protective role in developing psychological distress. Although the need to be rational can usually be understood as a maladaptive emotional schema when it blocks emotional processing, it may be helpful in some specific situations (da Silva et al., 2022) and can explain the result. These apparent contradictions reflect the need to understand emotional schemas within a specific context. A negative schema may be adaptive under particular circumstances or when referring to specific emotions.

To our knowledge, no other study has focused on different models for each parental figure to analyse emotional schemas’ mediation role. The literature suggests the relevance of paternal emotional invalidation on developing psychological distress (e.g., Lugo-Candelas et al., 2016; Ramirez, 2018). Our study appears to support this idea. The direct effect of recalled maternal, but not paternal, emotional invalidation on psychological distress was no longer significant when emotional schemas were considered. In other words, the role of maternal emotional invalidation in psychological distress seems to happen only through internal models. However, paternal emotional invalidation continues to have a direct effect on psychological distress even when emotional schemas are considered. This suggests that the role of paternal emotional invalidation on psychological distress may not occur only through internal models. Nevertheless, the results suggest that emotional schemas intervene in how parental emotion socialisation translates into psychological distress.

The major difference between models was observed related to the mediational effect of the need to be rational in the relationship between recalled emotion socialisation (validation and invalidation) and psychological distress, observed only in the father’ model. Studies with similar variables did not analyse the mother and father separately (Boucher et al., 2013; Dinis, 2014; Edwards et al., 2016; Krause et al., 2003; Sauer & Baer, 2009; Westphal et al., 2016). We recommend that future studies should explore these mediation relationships. Comparing the two models, the fathers’ model explains slightly more variability of emotional schemas and psychological distress. Although some authors advocate that the impact of mother and father on children’s development is very similar (Shewark & Blandon, 2015). Other authors (Cabrera et al., 2014; Shortt et al., 2016) argue that mothers and fathers can have different roles in developing emotional regulation and psychological distress. The present study is consistent with the notion of a differential role for each parent.

However, analysing all the mediators, the strongest mediator in the relationship between recalled emotional invalidation and psychological distress was the negative evaluation of emotion in both models. The strongest mediator in the relationship between recalled emotional validation and psychological distress was difficulties in reappraisal. It seems that, at least in a community sample, a negative evaluation of emotion is more important than the rest of the emotional schemas in the relationship between recalled parental emotional invalidation and psychological distress. Our study highlights the importance of parental emotional invalidation on developing negative evaluation of emotion and, in turn, psychological distress. This suggests that for individuals growing up with invalidating parents, a more cognitive dimension (negative evaluation of emotion) (da Silva et al., 2022) contributes more to the development of psychological distress. And the importance of parental emotional validation and its protective role in developing difficulties in reappraisal and, in turn, protecting the development of psychological distress. This suggests that for individuals who have grown up with parents who mostly validate and support their emotions, a more emotional, motivational, and behavioural dimension (difficulties in reappraisal) (da Silva et al., 2022) contributes more to protecting the development of psychological distress. In this respect, there seems to be a convergence in emotional socialisation between the two parental figures. This can also result from when individuals recall parental emotion socialisation during the first eighteen years of life; they remember and report more general descriptions and do not think separately of their father or mother.

The two models explain around 60% of the observed variance of psychological distress. Other variables not included in the models can be important to explain emotional schemas and psychopathological symptoms. Future studies could include attachment, partner emotion socialisation, and friends’ emotion socialisation because these variables appear important in developing emotional schemas (Leahy, 2019). It would be interesting to observe if emotional schemas continue to mediate the relationship between these variables and psychological distress. Studying the role of culture and parental style in the proposed models could also be important. Our study was conducted in a particular European context and can be compared to future applications in diverse cultural contexts.

These findings should be interpreted considering some limitations of the present study. Firstly, this study used cross-sectional data, which does not allow inferring causality in the studied relations. Secondly, the study was conducted with a convenience sample, which does not allow for generalising the results to the general population. Thirdly, the retrospective nature of the parental emotion socialisation measure represents another study limitation. Fourthly, self-report questionnaires were used to collect data simultaneously from the same participants. Fifthly, the sample included a large age range. Even considering that the analysis was controlled by age and gender, it begs the question of how life experiences could alter recalled parental emotion socialisation. Future studies could focus on analysing these relationships in the same generation. Sixthly, the sample was relatively small and was primarily constituted by individuals who grew up in traditional families with a low rate of divorces, which does not match the current Portuguese reality. Different types of families may influence the impact of parental emotion socialisation. Future studies could focus on these differences – single-parent families, same-sex parents, and reconstructed families.

## Conclusions

The present study has several theoretical and clinical implications. From a theoretical point of view, this study contributes to the progress of the emotional schema’s developmental perspective. It suggests that recalled parental emotional invalidation constitutes a risk factor for developing psychological distress. The present study considered two levels of explanation for psychological distress: distal causes (past parental socialisation) and proximal causes or maintenance factors (emotional schemas). This allows considering these two levels of explanation in understanding how parental emotion socialisation leads to psychological distress through emotional schemas.

The results may contribute to prevention and early intervention. It could be important to include in parenting skills training the emotionally adequate response to their children’s emotional expression, knowing how to teach and guide their emotional experience and adaptively expressing their emotions, promoting emotional validation experiences, and minimising invalidation experiences. These actions will allow parental emotion socialisation to be a protective factor against developing (maladaptive) emotional schemas. Additionally, the results may suggest the relevance of working on emotional schemas in psychotherapy. Although parental emotion socialisation cannot be changed in adults, psychotherapy can minimise its influence by targeting emotional schemas.

## Supplemental Material

Supplemental Material - Recalled Parental Emotion Socialisation and Psychological Distress: The Role of Emotional Schemas Supplemental Material for Recalled Parental Emotion Socialisation and Psychological Distress: The Role of Emotional Schemas by Rita Sebastião, David Dias Neto and Ana Nunes da Silva Emma Ballard in Psychological Reports

## Data Availability

The datasets generated during and/or analyzed during the current study are available from the corresponding author on reasonable request.
