# Supplementary material for: Recalled Parental Emotion Socialisation and Psychological Distress: The Role of Emotional Schemas
Source: Psychol Rep. 2023 Sep 28;128(5):3264–81. doi: 10.1177/00332941231204304 (PMC12394774; doi:10.1177/00332941231204304)
Supplement: Supplemental Material - Recalled Parental Emotion Socialisation and Psychological Distress: The Role of Emotional Schemas [file sj-pdf-1-prx-10.1177_00332941231204304.pdf]

Table S1

Father's and Mother's Models Total and Direct Effects: Standardised Estimates, Corresponding Standard Errors, and Significance

	β	*SE*	*p*
Father model			
Total effects			
Invalidation → Psychological distress	.37	.05	<.001
Validation → Psychological distress	-.20	.04	.002
Direct effects			
Invalidation → Psychological distress	.19	.05	.007
Validation → Psychological distress	.05	.04	.468
Invalidation → Negative evaluation of emotion	.38	.06	<.001
Invalidation → Difficulties in reappraisal	.04	.07	.555
Invalidation → Difficulties in naturalising emotion	.37	.07	<.001
Invalidation → Need to be rational	.23	.06	.002
Invalidation → Simplistic view of emotion	.05	.07	.534
Validation → Negative evaluation of emotion	-.24	.05	<.001
Validation → Difficulties in reappraisal	-.44	.06	<.001
Validation → Difficulties in naturalising emotion	-.008	.06	.912
Validation → Need to be rational	-.19	.05	.005
Validation → Simplistic view of emotion	.17	.06	.017
Negative evaluation of emotion → Psychological distress	.64	.15	.002
Difficulties in reappraisal → Psychological distress	.43	.09	.001
Difficulties in naturalising emotion → Psychological distress	-.03	.09	.781
Need to be rational → Psychological distress	-.32	.09	.003
Simplistic view of emotion → Psychological distress	.23	.08	.052
Mother model			
Total effects			
Invalidation → Psychological distress	.30	.05	<.001
Validation → Psychological distress	-.11	.04	.098
Direct effects			
Invalidation → Psychological distress	.13	.05	.089
Validation → Psychological distress	.10	.05	.180
Invalidation → Negative evaluation of emotion	.32	.06	<.001
Invalidation → Difficulties in reappraisal	.04	.06	.564
Invalidation → Difficulties in naturalising emotion	.29	.07	<.001
Invalidation → Need to be rational	.19	.07	.021
Invalidation → Simplistic view of emotion	.02	.07	.838
Validation → Negative evaluation of emotion	-.16	.06	.018
Validation → Difficulties in reappraisal	-.43	.07	<.001
Validation → Difficulties in naturalising emotion	.05	.07	.512
Validation → Need to be rational	-.13	.07	.093
Validation → Simplistic view of emotion	.19	.07	.011
Negative evaluation of emotion → Psychological distress	.69	.15	.001
Difficulties in reappraisal → Psychological distress	.42	.09	.001
Difficulties in naturalising emotion → Psychological distress	-.02	.09	.880
Need to be rational → Psychological distress	-.33	.09	.003

Simplistic view of emotion → Psychological distress	.20	.08	.091
